# Particulate Organic Matter Distribution along the Lower Amazon River: Addressing Aquatic Ecology Concepts Using Fatty Acids

**DOI:** 10.1371/journal.pone.0046141

**Published:** 2012-09-28

**Authors:** Jean-Michel Mortillaro, François Rigal, Hervé Rybarczyk, Marcelo Bernardes, Gwenaël Abril, Tarik Meziane

**Affiliations:** 1 UMR-CNRS-IRD-UPMC 7208, BOREA, département milieux et peuplements aquatiques, MNHN, CP 53, Paris, France; 2 Universidade dos Açores, Azorean biodiversity group, rua Capitão João d'Ávila, São Pedro, Angra do Heroísmo, Terceira, Portugal; 3 Universidade federal Fluminense, instituto de química, programa de geoquímica, Niteroi, Rio de Janeiro, Brasil; 4 UMR-CNRS 5805, EPOC, université Bordeaux 1, Talence, France; 5 IRD, GET, laboratorio de potamologia Amazônica, UFAM, avenida general rodrigo Octávio Jordão Ramos, Manaus, Amazonas, Brasil; Royal Netherlands Institute of Sea Research (NIOZ), The Netherlands

## Abstract

One of the greatest challenges in understanding the Amazon basin functioning is to ascertain the role played by floodplains in the organic matter (OM) cycle, crucial for a large spectrum of ecological mechanisms. Fatty acids (FAs) were combined with environmental descriptors and analyzed through multivariate and spatial tools (asymmetric eigenvector maps, AEM and principal coordinates of neighbor matrices, PCNM). This challenge allowed investigating the distribution of suspended particulate organic matter (SPOM), in order to trace its seasonal origin and quality, along a 800 km section of the Amazon river-floodplain system. Statistical analysis confirmed that large amounts of saturated FAs (15:0, 18:0, 24:0, 25:0 and 26:0), an indication of refractory OM, were concomitantly recorded with high pCO_2_ in rivers, during the high water season (HW). Contrastingly, FAs marker which may be attributed in this ecosystem to aquatic plants (18:2*ω*6 and 18:3*ω*3) and cyanobacteria (16:1*ω*7), were correlated with higher O_2_, chlorophyll *a* and pheopigments in floodplains, due to a high primary production during low waters (LW). Decreasing concentrations of unsaturated FAs, that characterize labile OM, were recorded during HW, from upstream to downstream. Furthermore, using PCNM and AEM spatial methods, FAs compositions of SPOM displayed an upstream-downstream gradient during HW, which was attributed to OM retention and the extent of flooded forest in floodplains. Discrimination of OM quality between the Amazon River and floodplains corroborate higher autotrophic production in the latter and transfer of OM to rivers at LW season. Together, these gradients demonstrate the validity of FAs as predictors of spatial and temporal changes in OM quality. These spatial and temporal trends are explained by 1) downstream change in landscape morphology as predicted by the River Continuum Concept; 2) enhanced primary production during LW when the water level decreased and its residence time increased as predicted by the Flood Pulse Concept.

## Introduction

Every year, the Amazon River and its tributaries, which together drain the Amazonian Basin, overflow and flood the adjacent forest, forming extensive wetlands [1]. All of these wetlands include a great variety of natural habitats, such as floodplains, which support the growth of aquatic organisms [2]. Floodplains, locally known as Várzea, are areas periodically inundated and oscillate between aquatic and terrestrial phases. On an annual basis, Várzea may account for a source of water to the Amazon River [3], [4], with up to 30% of water in the main river channel which passes through the floodplains [5]. Within the Amazon Basin, floodplains cover about 350,000 km^2^ [6] and may also constitute one of the major sources of organic matter (OM) to the Amazon River [7], [8]. There is still a general lack of understanding about global organic carbon dynamics at the world level, particularly due to the high degrees of spatial and temporal variabilities of OM sources in large river ecosystems [9]. In aquatic ecology, the ways in which OM is distributed in these ecosystems have been couched in a few hypotheses, including the River Continuum Concept (RCC) [10], the Flood Pulse Concept (FPC) in river-floodplain systems [11] and the Riverine Productivity Model (RPM) [12]. The RCC considers river/streams as a single ecosystem in order to predict the variability of biological communities and longitudinal changes from headwaters to river mouths, and it also emphasizes the import of allochthonous and autochthonous matter from upstream sections. However, according to the FPC, seasonal inundation, which has a structuring role for energy and nutrient dynamics in river-floodplain systems, increases productivity within the floodplain areas. Both concepts were challenged by the promoters of the RPM, which predicts that autochthonous production in the river channel provides a substantial portion of the organic carbon and lower contributions from floodplains and upstream sections. Typically, the Amazon Basin, with its geomorphology and the magnitude of hydrological fluxes within its channels, is a suitable system in which can be evaluated the validity of these concepts on OM origin and fate [13].

Composition and quality of suspended particulate OM (SPOM) in the Amazon Basin have been previously documented using stable isotopes [14], [15], fatty acids (FAs) [8], [16], amino acids and lignin phenols [15], [17]. The OM has been reported as refractory in the river [18], [19], whereas it has been described as more labile in the Várzea [14]. However, few studies have prospected OM composition and quality of floodplains and rivers of the Amazon basin together in the same survey. Moreover, we have a little knowledge about the spatial and temporal changes of OM composition and quality at a large scale [8].

Analysis of FA compositions in SPOM is commonly used to characterize the origin and fate of OM in freshwater ecosystems [8], [20], [21]. The FAs composition of OM, and the occurrence of specific markers, permit to define food web relationships in the water column [21]. The occurrence of specific markers permits also to identify allochthonous and autochthonous sources in an environment [22], [23]. Spatial and temporal variations of OM quality can also be reported [23]–[25]. Although FAs of SPOM were previously used in [8] to characterize sources of OM, the main purpose of this study was to see how spatial structuring of the Amazon River system can affect the FA composition of SPOM. More precisely, due to the isolation of Várzea during the low water season, one can hypothesize that floodplains would become incongruous from the main channel than during the flooding period. To the contrary, at high water season, large homogenization between Várzea and the rivers should occur in regards to the FPC [11] and should be also indicated by FA compositions of SPOM.

Consequently, the specific aims of the study were to 1) identify and quantify the spatial patterns of FAs composition in two marked seasons: high waters (HW) in June 2009 and almost low water (LW) in October 2009, 2) relate spatial and seasonal variability of OM quality to environmental variables in aquatic ecosystem, 3) discuss the validity of FAs as spatial and temporal predictors of OM quality and 4) address the probable origin and transfer of OM in large river-floodplain ecosystem, based on aquatic ecology concepts.

## Materials and Methods

### Study area

Geological formation of the Amazonian Basin resulted in the largest river system on earth, with 7,050,000 km^2^ of land drained toward the Amazon River, interlaced by numerous large and small rivers [1], [26]. The length of the Amazon was estimated to be ca. 7,000 km, reaching below the mouth of the Negro River a width of 4 to 5 km. The mean depth of the river main channel is between 40 and 50 m, reaching in places ca. 100 m, where the bottom lies deeper than sea-level in the deepest parts of the river-mouth [1], [27], [28].

**Figure 1:**
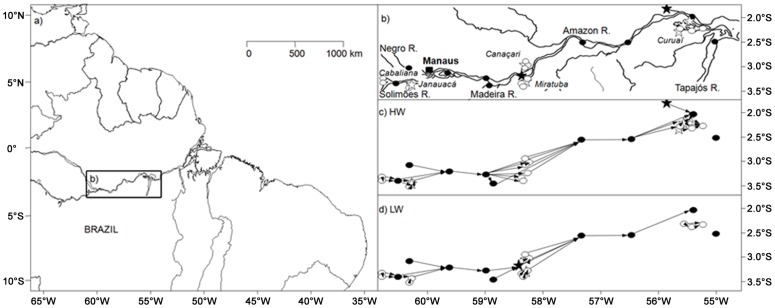
Location of the study area and connection diagram between sampled sites used to generate AEM and PCNM functions in both HW and LW. Study area (a) on the Amazonian Basin (Brazil). Framed area (b) highlight the different sampling sites, Várzea names are in italic. Connection diagrams (c, d) highlight the links between stations. Várzea stations (open circles), white, black or clear water rivers (closed circles), stations sampled in a single season (stars).

Samples were collected on a ca. 800 km transect along the Lower Amazon River from Manacapurú (3°18′30″S; 60°52′34″W) on the Solimões River, to Santarem (2°28′28″S; 55°0′56″W) at the mouth of the Tapajós River (Figs. 1a and 1b). Sampling sites were located in five rivers (Solimões, Negro, Madeira, Amazon and Tapajós, Fig. 1b) and in five floodplain lakes (Cabaliana, Janauacá, Canaçari, Miratuba and Curuaï, Fig. 1b). Samples were collected in June 2009 during the HW season and in October 2009, one month before the lowest water level (LW). As the Amazon hydrograph is relatively uniform from year to year [7], [29], these two sampling campaigns were assumed to constitute sufficient resolution to examine seasonal changes in OM composition.

### Sample collection and preparation

FAs data used in this study were obtained and chemically extracted from SPOM material sampled along the Amazon River and Várzea in 2009 as detailed in Mortillaro et al. [8]. Briefly, three replicates of surface SPOM were collected from each station with a Niskin bottle and filtered immediately through pre-combusted (450°C, 12 hours) glass fiber filters (Whatman GF/F, 47 mm diam.) using a vacuum system under low pressure. All samples were frozen (−20°C) on the research vessel and transported frozen to France for lipid analysis. Freeze dried samples were then processed following a slightly modified method of Bligh and Dyer [30] as in Meziane et al. [31]. FA mean concentrations (i.e. three replicates for each station, µg l^−1^, detection limit of 0.03% by weight of the original sample) were used to build FA-by-site matrix (hereafter the matrix **F**).

### Environmental variables

Environmental variables were measured on board during each cruise, with conductivity (µS cm^−1^, ±0.05%), pH, turbidity (nephelometric turbidity units, ±0.3 NTUs), and O_2_ (mg l^−1^, ±2%) using a multiparameter probe (YSI 600XLM). Sensors were calibrated each week, according to the manufacturer's instructions. Equilibrator system was used to measure *in-situ* CO_2_ partial pressure (pCO_2_, μatm) [32], [33]. Chlorophyll *a* (Chl *a*, µg l^−1^) and pheopigments (Phe, µg l^−1^), were measured on GF/F filters, according to the method described by Parsons *et al.* [34] using a 10-AU Turner Fluorometer (detection limit: 0.025 µg l^−1^). Dissolved organic carbon (DOC, mg l^−1^) was measured in the filtrates using a Shimadzu TOC-VCSH analyzer (detection limit, 20 µmol l^−1^). Total alkalinity (TA, mmol kg^−1^) was measured on filtrates by Gran electrotitration (±4 µmol kg^−1^) with 0.1 N HCl [35].

### Statistical analysis

A detailed graphical description of the analysis and the statistical procedure we adopted here is given in the Figure S1. The same pipeline of analyses was applied for the two seasons. The strategy was three-fold: 1) identify and quantify (i.e. in terms of percentage of variance explained) the spatial structure in the FA distribution; 2) evaluate the predictive power of environmental variables in the FA distribution; 3) estimate the independent and joint effect of both spatial and environmental variables in the FA distribution.

Prior to the statistical analyses, this **F** matrix was Hellinger-transformed to reduce the influence of extreme values [36]. To identify the most predominant spatial patterns, two eigenfunction-based spatial filtering approaches were employed. Spatial vectors were derived using: 1) principal coordinates of neighbor matrices (PCNMs) which is a well-suited method to detect spatial trends across a wide range of scales [37]–[39] and 2) asymmetric eigenvector maps (AEMs) which is mainly designed to assess spatial structures in flow system (i.e. asymmetric forcing process)[40], [41]. As recommended by Blanchet et al. [41], the use of both methods helps to better understand the spatial structure in the systems that are not fully directional [41].

**Figure 2:**
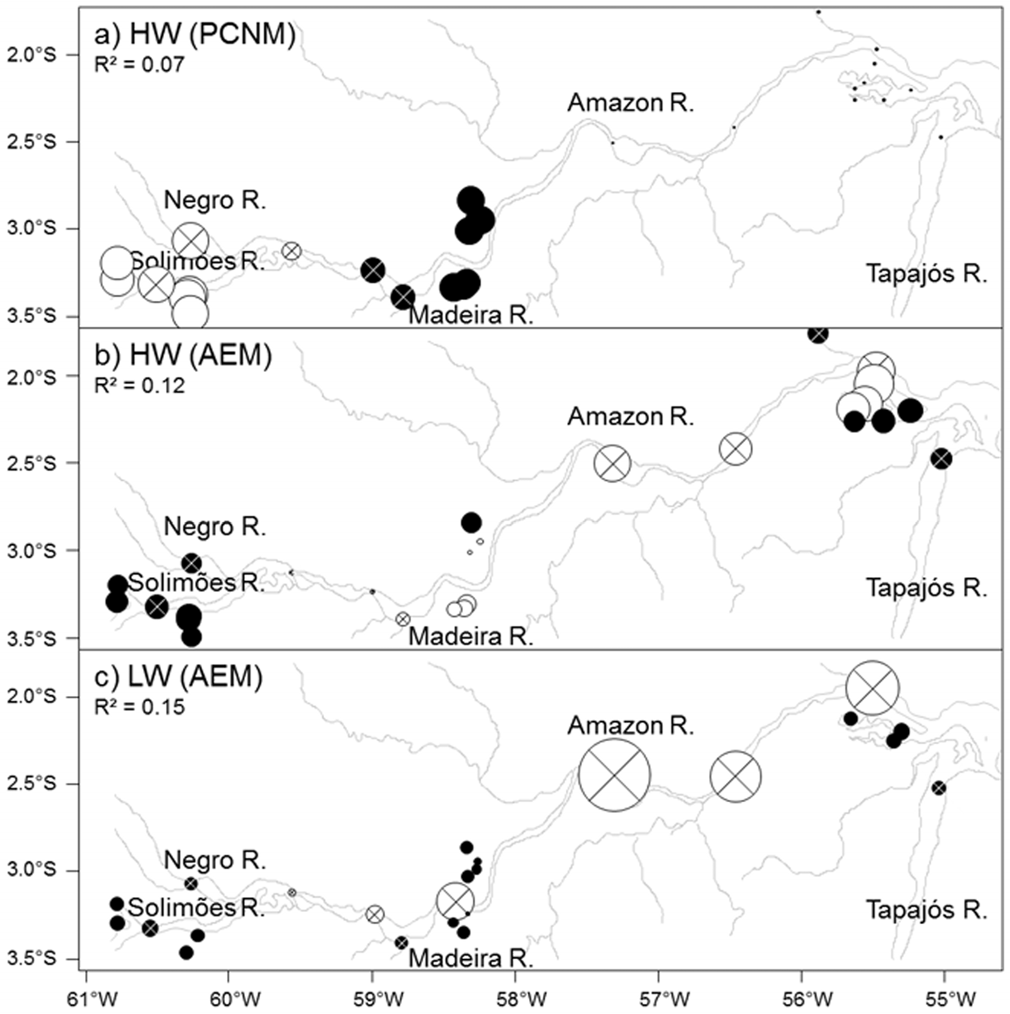
Spatial modeling of the fatty acids composition in the Amazon Basin for HW and LW. The PCNM and AEM eigenvector site scores selected by FS were mapped for HW in (a) and (b) and for LW in (c). Note that for LW, any PCNMs were significant. The bubbles represent the centered PCNM and AEM eigenvector site scores (mean = 0, standard deviation = 1) with positive (black circles) and negative (white circles) values proportional in area to the absolute value. Circles with cross are for river stations and without cross for Várzea stations. The percentage of variation (adjusted R^2^) explained by each eigenvector is given in parentheses.

The PCNM analysis describes spatial structures by quantifying the variability at all spatial scales. The PCNM method is based on a spectral decomposition of the study area into a series of eigenvectors each representing a spatial scale [37], [39]. AEMs decompose the spatial relationships among sampling sites into eigenvectors like PCNMs. However, AEMs reflect directional variations at specific spatial scales. AEMs were generated from a directional river network (Fig. 2) derived from satellite images where hydrological information was extracted [42], with empirical assumption of lateral and longitudinal connections between sampling sites, and from field observations. Further details about the PCNMs and AEMs implementation can be found in [37], [39], [41]. For both PCNM and AEM, Moran's *I* coefficient of spatial autocorrelation was computed to divide the set of spatial eigenvector generated in two groups, composed by the vectors displaying significant positive and negative autocorrelation. Both sets were tested using a Bonferonni correction to assess their global significance. Then, if a set was significant, a forward selection (hereafter FS, see Fig. S1a ; 9999 random permutations with a cut-off alpha of 0.05) was computed with the matrix **F** to keep the spatial vectors significantly correlated [43].

In order to avoid collinearity of environmental variables that can lead to misestimate model parameters and *R^2^* of canonical analyses, a principal component analysis (PCA) was carried out for each sampling season, and the five first PC axes that explained for both seasons 90% of the variance were retained. The variance percentages and variable contributions to the axes are given in Table 1. For each season, a FS was applied on the environmental PC axis to select the vectors (9999 random permutations with a cut-off alpha of 0.05) to be included 1) in a canonical redundancy analysis (RDA), 2) partial canonical redundancy analysis (pRDA) and 3) in a variation partitioning procedure [40]. RDA is a direct extension of the multiple regression analysis for the modelling of multivariate response data (i.e. a matrix of response variables and a matrix of environmental variables, Fig. S1b), whereas pRDA was used to estimate the single contribution of each selected PC axis with all other explanatory variables included as covariate. Variance partitioning led to split the variance of the response matrix into components explained solely by effects of environmental or spatial variables, components explained by combined effects of environmental and spatial variables, and finally unexplained components (Fig. S1c). PCNM and AEM vectors were included independently in the variation partitioning procedure [44]. Partitioning was carried out through a series of partial RDAs [45].

**Table 1:** Correlation matrix from the PCA of the environmental variables for HW and LW seasons.

	HW					LW				
	PCA1	PCA2	PCA3	PCA4	PCA5	PCA1	PCA2	PCA3	PCA4	PCA5
	(34.01)	(26.74)	(22.07)	(8.63)	(5.71)	(38.24)	(26.68)	(17.67)	(7.85)	(5.19)
conductivity	**−0,87**	0,34	−0,19	0	−0,17	*−0,61*	*0,4*	*−0,67*	0	−0,09
pH	**−0,88**	−0,21	−0,29	−0,18	−0,09	**−0,93**	−0,03	0,08	0,09	0,07
turbidity	*−0,48*	−0,18	0,37	**0,76**	−0,12	*−0,48*	*−0,49*	−0,32	*−0,57*	0,30
O_2_	−0,01	**−0,96**	0,07	−0,16	−0,11	**−0,74**	−0,12	*0,47*	0,38	0,13
CO_2_	0,18	**0,90**	−0,24	0,11	0,24	**0,89**	−0,01	−0,39	0,10	−0,10
TA	**−0,86**	0,36	−0,29	−0,12	−0,07	*−0,63*	0,39	*−0,61*	0,19	−0,14
Chl *a*	0,29	−0,25	**−0,90**	0,05	−0,1	−0,32	**−0,88**	−0,02	0,12	−0,20
Phe	0,34	−0,15	**−0,85**	0,32	−0,07	−0,05	**−0,90**	−0,15	0	−0,31
DOC	*0,56*	*0,52*	0,19	−0,08	*−0,61*	0,39	*−0,48*	*−0,51*	*0,41*	*0,42*

Values in brackets are the variance explained (eigenvalues) per each PCA axis. Values in italic *r>0.4* or *r<−0.4* (modest correlation, 0.001>*p*>0.05) and in bold **r>0.7** or **r<−0.7** (strong correlation, *p*<0.001).

Finally, the relative importance of spatially structured environmental variations and spatial effects alone on matrix **F**, at different scales, was assessed by building scale-specific additive spatial models (Fig. S1d) [46] for each selected spatial eigenvector (i.e. each spatial scale). This procedure allowed analyzing the relative significance of spatially structured environmental components at the specific scales of PCNM and AEM variables independently of FA assemblages.

All the *R^2^* values provided by the analysis were adjusted to account for the number of sampling sites and explanatory variables, as unadjusted *R^2^* values are biased. All the statistical analyses conducted in this study were implemented within the R programming environment (R Development Core Team 2010) using the packages ‘vegan’ [47] for variation partitioning and PCNMs, the package ‘packfor’ [48] for the FS of explanatory variables and the package ‘rdaTest’ for the RDA and pRDA [49]. AEMs were extracted using the AEM package [50].

## Results

### Spatial distribution of FA variables

Figures 1c and 1d present a schematic map of the stations network in the study area, illustrating the decrease of connections between lakes and rivers during the falling water level (LW) compared to HW. AEM and PCNM spatial methods were then used for both seasons in order to report the prevalent spatial structure in these networks (Fig. 2). Among the set of spatial eigenvectors produced by the AEM procedure (i.e. 20 and 16 for HW and LW seasons, respectively), only one was selected for each season by FS (*p*<0.001). For both seasons, the selected eigenvectors illustrated discrimination between the Amazon River and lakes, which discrimination appears more contrasted during LW (Figs. 2b, c). Among the spatial eigenvectors produced by the PCNM procedure (i.e. 12 for both seasons), only one was selected during HW by FS (*p* = 0.009), whereas no eigenvector was significant for LW season (*p*>0.05). This selected spatial eigenvector for HW described an upstream-downstream gradient on the basin (Fig. 2a).

### Environmental contribution to FA variables

**Figure 3:**
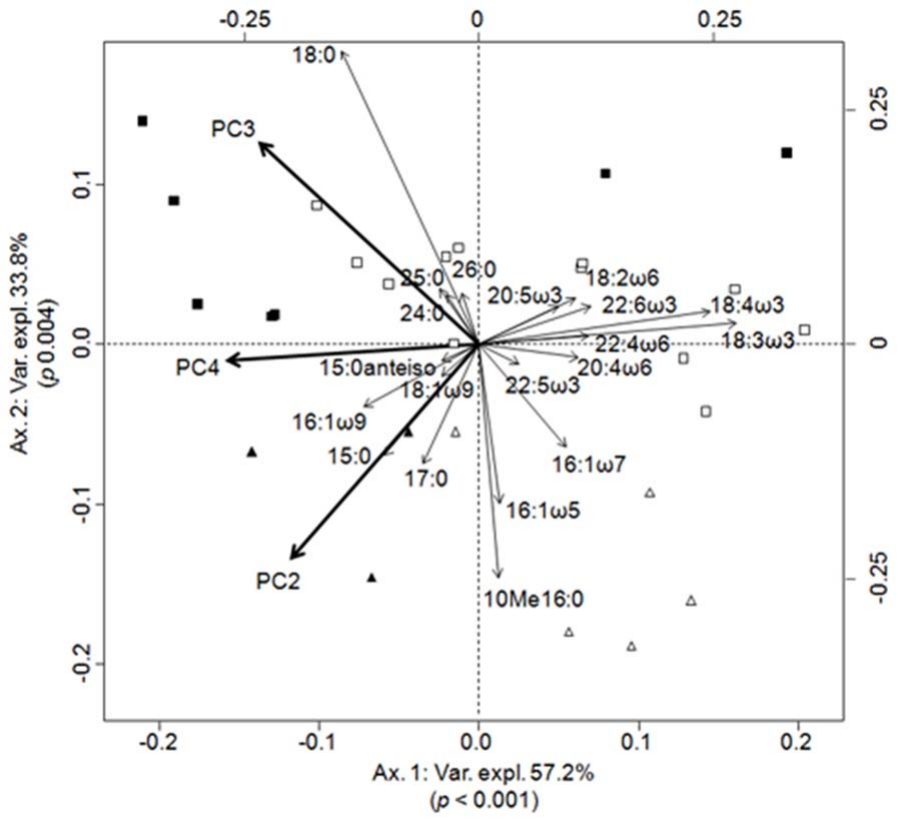
Biplot of canonical redundancy analyses between FAs composition per site and environmental variables during HW. The FAs most correlated to the first two canonical axes are shown for an easy-reading of the figure. Downstream (triangles) and upstream (squares) stations are according to PCNM map (Fig. 3a). Várzea (open), rivers (closed). The angles between variables in the RDA biplot reflect their correlation coefficient.

PCA output is summarized in Table 1, which focuses on the correlation of each environmental variable to PC axes. For HW, three PC axes (PC3, PC2 and PC4) were selected by FS while five PC axes were retained for LW (Table 2). In HW, PC3 (negatively correlated to Chl *a* and Phe), PC2 (positively and negatively correlated to O_2_ and pCO_2_, respectively) and PC4 (positively correlated to turbidity) accounted together for 20% of variance explained by the environment (adjusted R^2^, RDA, F = 3.298, *p*<0.001 see Table 2 for the contribution of each PC axis). The biplot of the RDA is presented in Figure 3. The three PC axes from HW are correlated with river stations (i.e. on the left-side of the biplot) and hence illustrates the turbid aspect of rivers (PC4) with high pCO_2_ (PC2), as well as the weak concentration of Chl *a*, Phe (PC3), and O_2_ (PC2). In addition, the PC axes of the RDA biplot from HW are correlated to FA assemblages related to bacteria, detrital material, and vascular plants mainly composed of 15:0anteiso, 15:0, 16:0iso, 16:1*ω*9, 18:0, 18:1*ω*9, 24:0, 25:0 and 26:0. The right-side of the biplot is correlated to mono (MUFA) and polyunsaturated FA (PUFA) 16:1*ω*7, 18:2*ω*6, 18:3*ω*3, 18:4*ω*3, 20:4*ω*6, 20:5*ω*3, 22:4*ω*6, 22:5*ω*3 and 22:6*ω*3 which suggests a fresher OM such as from phytoplankton and/or macrophytes in Várzea stations. However, Várzea stations are spread in both sides of axis one (Fig. 3) which suggest a disparity in primary production.

**Table 2:** Results of the partial canonical redundancy analysis (pRDA).

	PC axes	*F*-ratio	*P*-values	*R^2^* (%)
HW season	PC3	3.50	0.008**	10.73
	PC2	3.17	0.003**	9.93
	PC4	2.88	0.012*	8.83
LW season	PC2	5.47	<0.001***	15.14
	PC1	3.51	0.006**	10.86
	PC4	2.10	0.053	7.82
	PC3	2.14	0.031*	6.31
	PC5	2.14	0.034*	6.03

P-values are tested with 9999 permutations tests. *P*-*values* *<0.05, **<0.01, ***<0.001. Semi-partial R^2^ are given.

**Figure 4:**
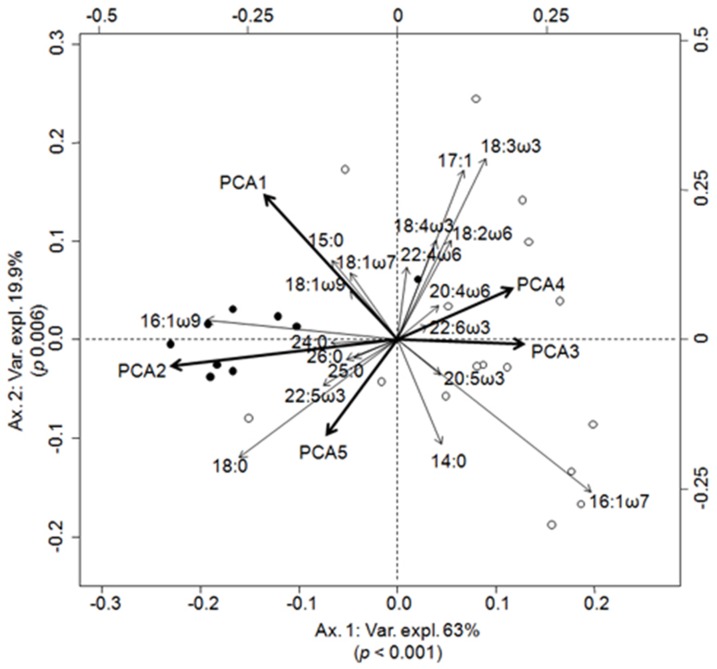
Biplot of canonical redundancy analyses between FAs composition per site and environmental variables during LW. The FAs most correlated to the first two canonical axes are shown for an easy-reading of the figure. Várzea (open), rivers (closed). The angles between variables in the RDA biplot reflect their correlation coefficient.

In LW, the PC2 axis has a main negative correlation to Chl *a* and Phe (Table 1), which with the PC1 (pH, O_2_ and pCO_2_), PC3 (mainly conductivity), PC4 (mainly turbidity) and PC5 (mainly DOC) accounted together for 32% of the variance explained by the environment (adjusted R^2^, RDA, F = 3.207, *p*<0.001, see Table 2 for the contribution of each PC axis). The biplot of the RDA highlights discrimination between the Amazon River and lakes (Fig. 4). PC2 and PC1 are correlated to the Amazon River (Fig. 4, left-side of the RDA biplot) meaning that the latter was related to high pCO_2_ and low Chl *a*, Phe, and O_2_, conversely to the lakes (Fig. 4, right-side of the RDA biplot). In addition, lakes are correlated to FA assemblages 16:1*ω*7, 18:2*ω*6, 18:3*ω*3, 20:4*ω*6 and 22:6*ω*3 usually found in primary producers as microalgae and C_3_ or C_4_ plants.

### Variation partitioning

**Figure 5:**
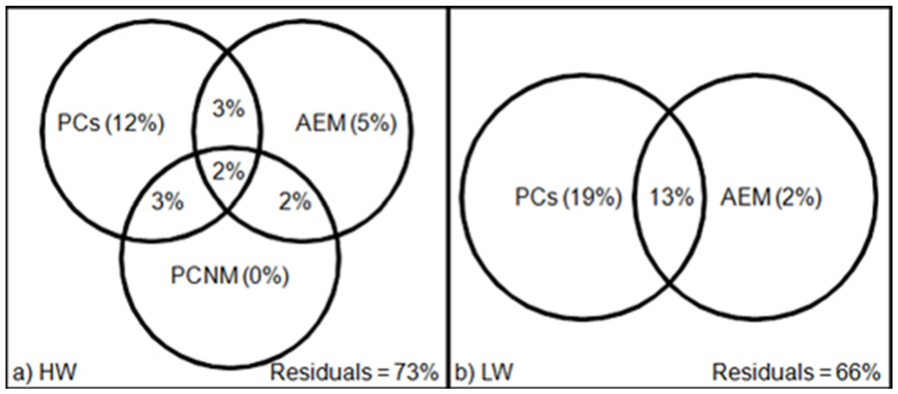
Venn diagram showing the results of the variation partitioning procedure. The variance partitioning was carried out on the forward selected environmental (PCs) and spatial variables (PCNM and AEM eigenvectors for HW and AEM for LW).

Variation partitioning revealed that during HW, 27% of the FA variance among SPOM samples was explained by environmental variables and spatial structures from the AEM and PCNM models, in which 12% was explained exclusively by environmental variables, 5% by the spatial structure found with the AEM, 8% by the combined effect of the environmental variables and space, and 2% of variance shared between AEM and PCNM eigenvectors (Fig. 5a). In LW, 34% of the FA variability of SPOM sampled was explained by environmental variables and spatial structure from the AEM model. These 34% were divided into 19% of exclusively environmental variables, 2% from the spatial structure given by the AEM, and 13% by the combination of spatially structured environmental variables (Fig. 5b).

**Figure 6:**
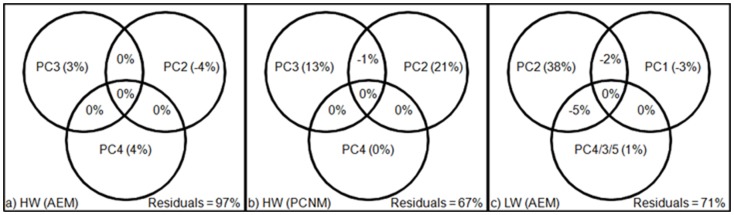
Venn diagrams showing the results of the specific additive model. The specific additive model was carried out on the environmental variables. a) AEM eigenvector for HW, b) PCNM eigenvector for HW and c) AEM eigenvector for LW.

The specific additive model applied for both seasons allowed dissecting the variance explained by environmental variables for each spatial structure independently. For HW, the additive model showed the null contribution of O_2_ and pCO_2_ (PC2) for the AEM spatial structure (Fig. 6a) as well as the weak contribution of the Chl *a*, Phe (PC3) and turbidity (PC4). In the opposite, for the PCNM spatial structure (Fig. 6b), O_2_ and pCO_2_ contributed to 21% of the variance (PC2), Chl *a* and Phe for 13% (PC3), and the null contribution of turbidity (PC4). For LW, Chl *a* and Phe (PC2) mainly accounted for 38% of the variance for the AEM spatial structure (Fig. 6c). It should be noticed that negative effects were observed for some variables and intersections which indicates synergistic effects, meaning that the variables together explained the variables response better than the sum of the individual effects [45].

## Discussion

FAs are generally used at small spatial and temporal scales as they are difficult to interpret as qualitative markers [23], [25], [51]. However, FAs are known to be adequate qualitative markers of OM in aquatic environment where sources are well defined, as it has been demonstrated in the Amazon basin for a first attempt [8]. Therefore, FAs of SPOM were to our knowledge, implemented for the first time into a large scale (800 km) multivariate analysis, to quantify the contribution of environmental and spatial variables to the quality of SPOM.

### SPOM quality and distribution

The RDA analysis (Figs. 3 and 4) indicated that during both seasons, saturated (SFAs, 15:0 and 18:0) and long chain FAs (LCFAs, 24:0, 25:0 and 26:0) were positively correlated with pCO_2_ and turbidity in the river (Figs. 3 and 4, Table 1). Correlation of these environmental variables with such FAs can confirm and strengthen the degraded status of OM in the river, as previously evidenced in Mortillaro et al. [8]. Indeed, unsaturated FAs, such as MUFAs and PUFAs, are rapidly degraded in aquatic ecosystems [16], [52], whereas SFAs and LCFAs are more resistant to degradation than short chain moieties [53]. Thus, FA compositions of OM indicate its degraded status in the Amazon River, as it was also reported by Aufdenkampe et al. [19], who used elemental (%OC, %N, C/N), isotopic (^13^C, ^15^N), hydrolysable amino acid and lignin phenol signatures, and by Hedges et al. [17], who used carbohydrates and amino acids, to assess the quality of OM.

Contrastingly, SPOM from floodplains was characterized in both seasons by FAs such as 16:1*ω*7 and 18:2*ω*6+18:3*ω*3 (RDA, Figs. 3 and 4), previously attributed to cyanobacteria [8], [54], [55] and aquatic plants, respectively [8], [56], [57]. However, these FAs may be shared with other organisms, and should not be used as unique markers. Therefore, correlation between MUFAs, PUFAs and high concentrations of O_2_, Chl *a* and Phe (Figs. 3 and 4, Table 1) confirms the occurrence of the primary production in Várzea. As indicated by the rapid degradation of unsaturated FA, the correlation between high concentrations of these FAs, O_2_, Chl *a* and Phe suggest that primary producer such as cyanobacteria and aquatic plants, supplies OM in Várzea. Therefore, from the present results, CO_2_ degassing in the river may result from a degradation process of fresh OM, which may originate from Várzea, than fuelled directly by degraded OM, which has been estimated to be 80% from terrestrial origin in the Amazon River [58].

For HW, the single significant AEM eigenvector allowed to distinguish Várzea sites from the Amazon River (Fig. 2b). However, this eigenvector was weakly responding to environmental variables (Fig. 6a). An upstream-downstream gradient in FA compositions of SPOM, generated by PCNM analysis, was also recorded during HW (Fig. 2a). This PCNM structure was correlated to pCO_2_ in downstream river stations, to O_2_ in upstream Várzea and to Chl *a* and Phe in downstream Várzea (PCs 2 and 3, Figs. 3and 6b). This correlation of the PCNM structure to environmental variables, suggests that despite the seasonal flood of the Amazon River, spatial distribution and quality of SPOM during HW were not uniform. Such an organic gradient was previously observed on several size fractions of OM [59], with an increasing degradation status of OM in downstream river stations.

During LW, only the eigenvector displayed by the AEM analysis was significant. The specific additive model indicated that Chl *a* and Phe (38%, Fig. 6c) were the main contributors to this eigenvector. This AEM eigenvector and the Chl *a* and Phe variables suggest therefore that during LW, primary production from the Várzea [8] had a predominant impact in structuring the SPOM distribution of this large-river ecosystem. This primary production in Várzea may have further implication, supporting food web dynamics in this ecosystem as suggested for larval fish production [60] and bacterioplankton [61]. It may also impact fisheries production as >40% of commercial fish receive most of their energy from planktonic algae [62]. Nevertheless, a large percentage of the FA variance in the SPOM composition remains unexplained. The unexplained variance may be the result of the variable itself. Indeed, SPOM was characterized by up to 50 individual FAs which together increase the heterogeneity among this biological variable. In addition, unexplained variance may be weakened adding potential relevant explanatory variables to our analysis. For instance, descriptors of the basin geomorphology are known to influence hydrodynamics [63], which in turn impact spatial composition of the SPOM [64]. Also, current velocity is likely to influence the distribution of SPOM which is transported over long distances in the Amazon River [1], [28].

### SPOM transfer and validation of aquatic ecology concepts

The RPM concept has been tested for the first time in a constricted region of the Ohio River [65]. This concept stresses the influence of autochthonous production and direct organic inputs from riparian zones into large rivers [12]. In the Amazon River, OM was mainly correlated to allochthonous detritic FAs (Figs. 3 and 4) and characterized as heterotrophic from environmental variables (Figs. 3 and 4) as expected for most rivers from their CO_2_ partial pressure [66], [67]. Thus, the deficiency of autochthonous productions in both seasons was confirmed in the river main channel [8] and was due to convergent effects of a shallow euphotic depth, deep water column and intense vertical mixing [1]. Therefore, the absence of autochthonous production in the rivers leaves the RPM concept unverified.

The RCC concept postulates that river networks are longitudinally linked systems in which downstream biotic assemblages and processes are linked to those of upstream parts [10]. During HW, spatial structure from the PCNM eigenvector indicated an upstream-downstream gradient (PCNM and RDA, Figs. 2a and 3). Thus, upstream stations were correlated to MUFA and PUFA, which suggest that OM in these stations was more labile than downstream (Fig. 3). This gradual change in OM composition could be related to decrease of forested surface cover from upstream to downstream [68]. Downstream decline of OM lability in the Amazon River can also be attributed, to alternate storage and retention phases in floodplains depending on the geomorphology of the basin [69]. However, the correlation of 16:1*ω*7, Chl *a* and Phe with downstream Várzea (Fig. 3, Table 1) suggests functional differences in primary production and supply during HW. Indeed, differences in maximum floodable area, depth and upland basin surface may occur throughout the study area [70]. These differences may impact the relative mixture of local and river water present in the Várzea [70] thus affecting the OM composition.

In river-floodplain systems such as the Amazon, the RCC must be combined with the FPC [11] to account for the lateral dimension including the Várzea. Indeed, these floodplains are dependent upon water exchanges with the Amazon River, which varies according to water level [3], [4], [63]. During LW, when floodplains are less connected to the Amazon River [3], [4], high concentrations of 16:1*ω*7, 18:2*ω*6 and 18:3*ω*3 were recorded in the Várzea as well as high values of Chl *a*, Phe and O_2_. These high concentrations explain spatial discrimination between Várzea and the Amazon River at this season (AEM, Fig. 2c), where a longitudinal gradient was absent (no eigenvectors found in the PCNM, nor displayed by the AEM). Maximum phytoplankton density was previously recorded during an isolation phase, which corresponds to our LW period [71], [72]. This maximum density may be related to the availability of nutrients [73] from the flood of the Amazon River three months earlier [74], but also from an increase of nutrient availability due to bottom sediments resuspension which occurs in Várzea during the isolation phase [3], [75]. Indeed, within the Várzea, sediment resuspension is induced by wind waves favored by a large fetch, regularly observed in white water lakes during LW while they reach their shallowest depth [3], [75]. This sediment resuspension also caused increase turbidity while residence time increase [75]. In this turbid environment, cyanobacteria may have a competing advantage, as they are able to migrate close to the water surface to reach their optimal photosynthesis light conditions [76], [77]. These issues, combined with competition and predation on phytoplankton, are the main drivers of the dynamics of these microorganisms in tropical floodplains [71], [72]. Therefore, according to the flood pulse concept, high resuspension of sediments and increased residence time into the Várzea prompted the observed autochthonous primary production in this aquatic ecosystem during the isolation phase.

## Conclusions

The OM characterization within the complex river-floodplain system remains a critical challenge for ecologists. OM does indeed play a key role in the Amazonian ecosystem, by structuring the food web [78] and by contributing to the carbon budget of the River [79]. The present study brought new insights on how 1) OM is spatially and seasonally structured, 2) Várzea is a hotspot of production during LW and consequently a source of presumably fresh SPOM for much of the system, and 3) seasonal water movements is a way to redistribute this fresh SPOM in the hydrologic network *via* the transfer to the river main channel. We concomitantly confirmed that FAs are adequate markers of the SPOM in this remarkable hydrodynamic environment.

## Supporting Information

Figure S1 Diagram showing step-by-step statistical methodology (see Material and Methods). In a) for the PCNM, d*ij* is the distance between sites *i* and *j*; max, maximum distance between two successive sites; PCoA, principal coordinate analysis, FS, forward selection allowing the selection of significant eigenvectors and PC axes and PCA, principal components analysis.
(DOC)
